# 567 Features of Burn Injuries in Youth: A Single Center Experience

**DOI:** 10.1093/jbcr/irac012.195

**Published:** 2022-03-23

**Authors:** Burak Ozkan, Ayse Ebru Abali, Murathan Erkent, Cem Aydogan, Gokhan Moray, Mehmet Haberal

**Affiliations:** Baskent University, Ankara, Ankara; Baskent University, Ankara, Ankara; Baskent University, Ankara, Ankara; Baskent University, Ankara, Ankara; Baskent University, Ankara, Ankara; Baskent University, Ankara, Ankara

## Abstract

**Introduction:**

Youth is categorized as combination of adolescence and young adulthood. Young people are prone to injuries, because this period involves essential changes in many aspects of life within a complicated physiological and mental developmental age. Our aim was to investigate the features of burn injuries in youth.

**Methods:**

Subjects were 622 adolescents (10-18yrs, n=313) and young adults (19-24yrs, n=309) who were treated at our burn-center from 2010 to 2021. Data collected for each case were age, sex, social-security status, occupation, marital status, scene of injury, burn extent, burn cause, history of injury, affected major body sites, and need for inpatient-care (median±SD,mean±SD)(p<.05).

**Results:**

Median age was 18yrs±4,76. Male to female ratio was 0,66:1 with female predominance especially among young adults (0,48:1)(p˂.05). Most subjects were in civil social-security-system (n=612, 98,4%); most were students (n=475, 76,4%); a total of 103 subjects were in labour-force(16,5%); 44 were unemployed(7,1%). Most young people were single (n=600, 96,4%); 4 subjects in adolescent group and 18 subjects in young adult group were married(0,64% and %2,9 respectively); 15 of married cases were female(68,2%)(p< .05). Injuries occurred mostly at home (n=411, 66,1%). Mean total body surface area (TBSA) burned was 3,21 % ± 7,5(min:0,2 max:75). The most commonly affected body site was the hand (n=165, 26,5%). Leading burn cause was scalds (n=433, 69,6%). Female subjects mostly suffered from scalds with mean TBSA burned of 2,0%±2,21 (min:0,2 max:18)(n=297, 79,2%). However, vast majority of flame burn victims were male (n=41, 82,0%) (mean TBSA burned: 12,8%±21,35, min:0,2 max: 75) and almost all severe electrical injuries happened to male subjects (n=13, 86,7%)(mean TBSA burned:14,2±14,81, min:0,2 max:40)(p˂.05). All cases were preventable accidents; the unique instance for ‘substance-abuse related burns’ was butane-lighter-liquid burns in 9 cases(1,4%). Inpatient-care was needed for 52(8,2%) victims. Mean TBSA burned for inpatients was 13,5%±19,6(min:1, max:75). All subjects survived.

**Conclusions:**

Our results suggest that young female subjects are prone to burn injuries, but severe injuries happen to male and there are many other aspects that should be considered. Combined evaluation of adolescents and young adults may provide purposive data for burn repositories.

